# Complete mitochondrial DNA sequence of *Tupaia belangeri yaoshanensis* (Wang, 1987) from Dayao Mountains in China

**DOI:** 10.1080/23802359.2023.2186723

**Published:** 2023-03-13

**Authors:** Yingying Cao, Shanshan Zhai, Zhuxin Li, Zhengtuan Huang, Liang Liang, Junyu Tao, Jian Xiao, Jing Leng, Haibo Tang

**Affiliations:** a Guangxi University of Chinese Medicine, Nanning, China; b Guangxi Key Laboratory of Translational Medicine for Treating High-Incidence Infectious Diseases with Integrative Medicine, Nanning, China; c Key Laboratory for Complementary and Alternative Medicine Experimental Animal Models of Guangxi, Nanning, China

**Keywords:** *Tupaia belangeri yaoshanensis* (*T.b. yaoshanensis*), mitochondrial genome, phylogenetic analysis

## Abstract

The tree shrew (*Tupaia belangeri*) is currently placed in the order Scandentia. Owing to their unique characteristics, such as small body size, high brain-to-body mass ratio, short reproductive cycle and life span, and low maintenance costs in laboratory conditions, tree shrews have been proposed as alternative experimental animals to primates in biomedical research. In this study, we determined the complete mitochondrial genome of the subspecies *Tupaia belangeri yaoshanensis* (*T.b. yaoshanensis*). The mitochondrial DNA (mtDNA) is 16,777 bp long and contains 13 protein-coding genes (PCGs), two ribosomal RNA genes (12S and 16S), and 22 transfer RNA (tRNA) genes. The base composition of the mitogenome was A (32.28%), T (26.82%), G (14.79%), and C (26.11%). For the 13 PCGs, 1405 variable sites were found between *T.b. yaoshanensis* and *T.b. chinensis* (JN800724), of which 916 were synonymous and 489 were nonsynonymous. The frequency of mutations significantly varied among the different genes, with the highest value in the *mt-NAD5* gene of tree shrews. Phylogenetic analysis based on the amino acid sequences of 13 PCGs revealed a closer relationship between the species of Scandentia and Lagomorpha. To the best of our knowledge, this is the first study to report the complete mitochondrial genome sequence of *T.b. yaoshanensis*.

## Introduction

**Figure 1.**
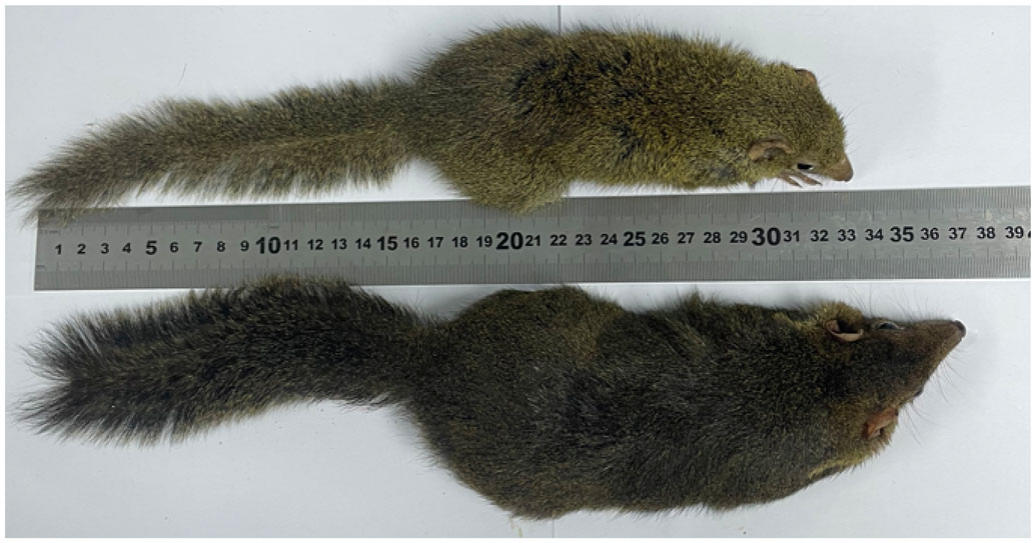
Photograph of *T.b. chinensis* and *T.b. yaoshanensis. T.b. chinensis* is located above the ruler, and *T.b. yaoshanensis* is located below the ruler. This picture was taken by the authors.

The tree shrew (*Tupaia belangeri*) is currently placed in the order Scandentia and is found throughout Southeast Asia and Southwest China (Wang 1987; Helgen 2005; Xu et al. 2012). This species exhibits unique characteristics that render it a potentially advantageous animal model, including a small body size, short reproductive cycle and life span, ease of handling, low maintenance costs in laboratory conditions, and genetic closeness to primates (Yao 2017). Wang (1987) suggested that Chinese tree shrews could be classified into six subspecies according to their geographical, morphological, and morphometric data (*Tupaia belangeri chinensis*, *Tupaia belangeri gaoligongensis*, *Tupaia belangeri modesta*, *Tupaia belangeri tonquinia*, *Tupaia belangeri yunalis*, and *Tupaia belangeri yaoshanensis*), marking the first attempt at subspecies classification for *Tupaia belangeri. Tupaia belangeri yaoshanensis* (hereafter, *T.b. yaoshanensis*) is a unique tree shrew subspecies inhabiting the Dayao Mountains of Guangxi, China (106°38′E, 24°63′N), in the northwestern part of the province. It has the largest body shape among tree shrews in China, with a weight of approximately 180 g and body length of approximately 200 mm (Figure 1). Previous studies have conducted mitochondrial genome sequencing of other tree shrew subspecies (accession nos.: JN800722–JN800724, NC_002521, Xu et al. 2012), but that of *T.b. yaoshanensis* has not yet been reported. We determined the complete mitochondrial genome of *T.b. yaoshanensis* to obtain more information regarding its phylogenetic status.

## Materials

The specimen was provided by the Experimental Center of Artificial Domestication and Cultivation of Guangxi University of Chinese Medicine. The specimen and DNA were stored at Guangxi University of Chinese Medicine, under voucher no. YS062-02 (Dr. Haibo Tang, tanghb@gxtcmu.edu.cn).

## Methods

Total genomic DNA from the liver tissue sample of *T.b. yaoshanensis* (no. YS062-02) was extracted using the phenol–chloroform extraction method (Green and Sambrook 2012). Based on the whole mitochondrial genome sequence of *T.b. chinensis* published in NCBI (accession no. JN800724, Xu et al. 2012), we used NCBI Primer-BLAST to design 14 pairs of primers that cover the whole mitochondrial DNA (mtDNA) (Table S1). The PCR products were purified on spin columns and sequenced using an ABI3730 Sequencing System (Sangon Biotech, Shanghai, China). The 14 sequences were spliced into a complete mitochondrial genome using DNAStar with the ‘Seqman’ function and finally submitted to GenBank (accession no. OP210313). The complete mitochondrial genome was annotated using the MITOS server (Bernt et al. 2013). Amino acid sequence homology alignment was performed using DNAStar with the ‘MegAlign’ function. Maximum-likelihood (ML) phylogeny was built using MEGA 7 with 1000 bootstrap replicates (Kumar et al. 2016; Nam et al. 2021).

## Results

**Figure 2.**
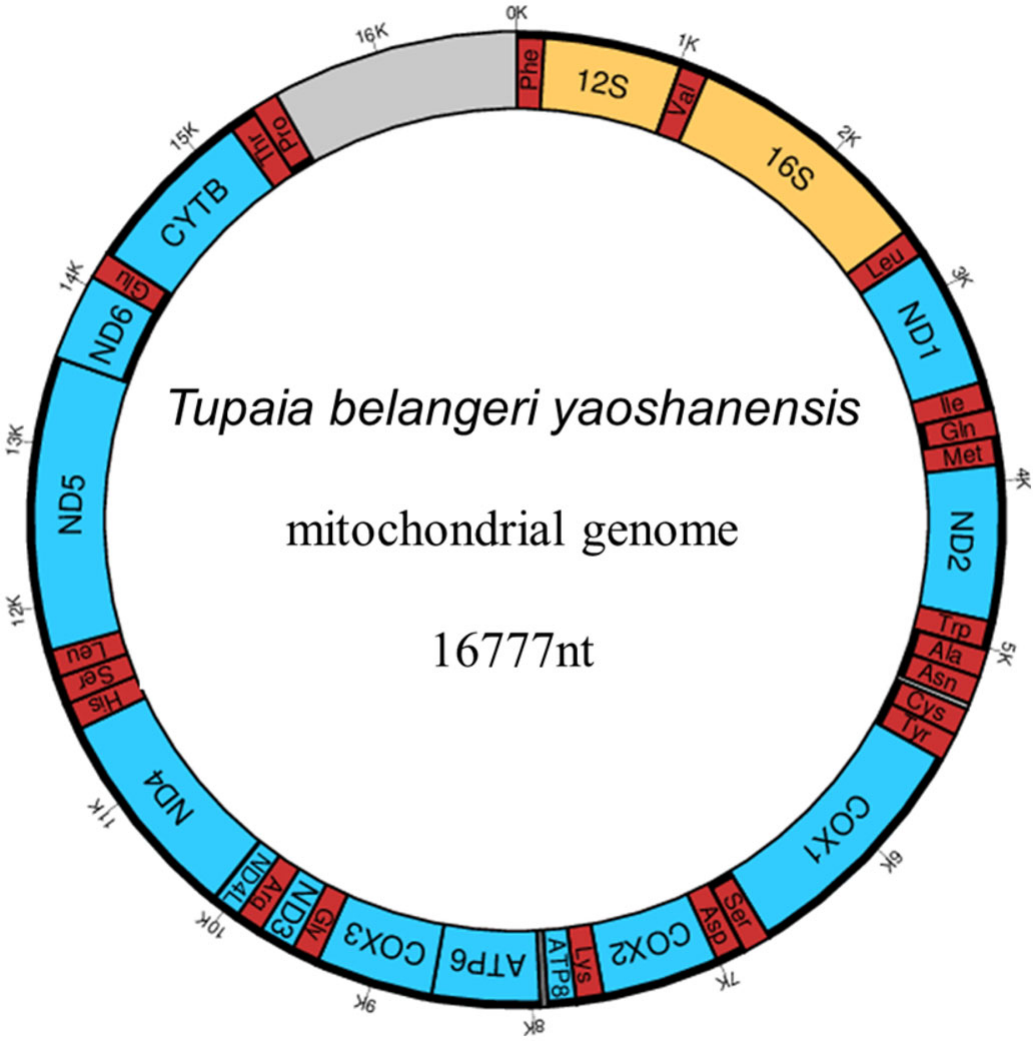
Gene map of the *T.b. yaoshanensis* mitochondrial genome.

The total length of the *T.b. yaoshanensis* mitochondrial genome was 16,777 bp with a GC content of 40.9% (Figure 2). The nucleotide composition was 32.28% A, 26.82% T, 14.79% G, and 26.11% C. The gene composition of the *T.b. yaoshanensis* mitogenome was 13 protein-coding genes (PCGs), two ribosomal RNA genes (12S and 16S), and 22 transfer RNA (tRNA) genes. For the 13 PCGs, a total of 1405 variable sites were found between *T.b. yaoshanensis* and *T.b. chinensis* (JN800724, Xu et al. 2012), of which 916 were synonymous and 489 were nonsynonymous. For amino acid sequence homology analysis, the 13 PCGs of tree shrews (*T.b. yaoshanensis*, *T.b. chinensis*) were 74.8–75% homologous to human PCGs, which is higher than those of mice (71.9%) and rabbits (74.6%). The mutation frequency varied significantly among the different genes, with the highest value observed in the *mt-NAD5* gene of tree shrews. For amino acid sequence homology analysis, the mt-NAD5 of tree shrew (*T.b. yaoshanensis*, *T.b. chinensis*) was 68.7–69.7% homologous to human mt-NAD5, which is higher than that of mouse (65%) and rabbit (67.9%). Furthermore, *T.b. yaoshanensis* mt-NAD5 had the highest amino acid sequence identity with humans (69.7%).

**Figure 3.**
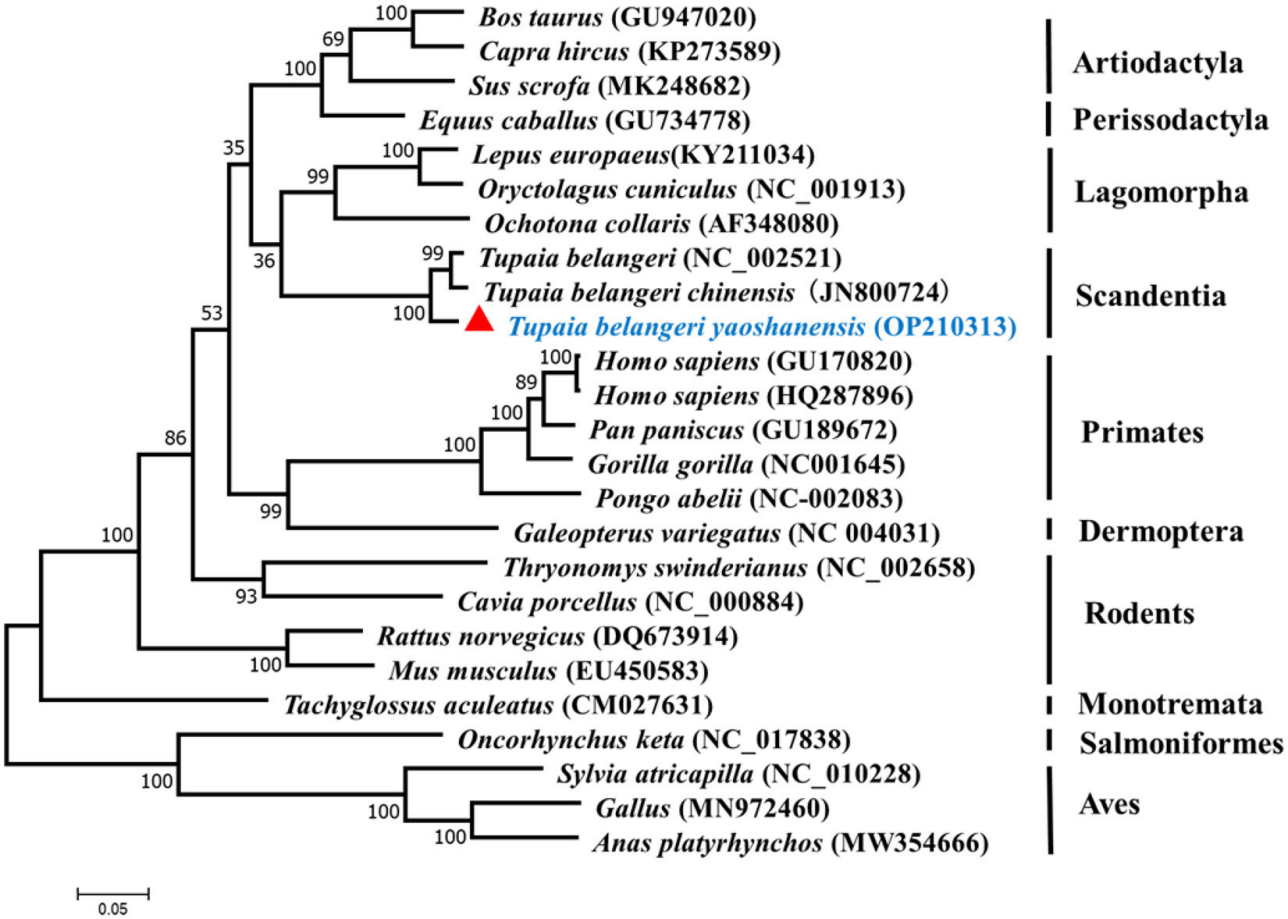
ML phylogenetic tree of 25 species developed based on 13 concatenated PCGs. Sequences of the 13 concatenated PCGs of *T.b. yaoshanensis* from southern China indicated by red triangles (▲). The nucleotide sequences of other 13 concatenated PCGs used in this study were obtained from GenBank.

Phylogenetic analysis was inferred from the available mtDNA of *T.b. yaoshanensis*, other tree shrew subspecies, primates, rodents, lagomorpha, artiodactyla, perissodactyla, dermoptera, monotremata, salmoniformes, and aves based on the amino acid sequences of 13 PCGs (for accession numbers, see Figure 3). Phylogenetic analysis showed that *T.b. yaoshanensis* was clustered with *T. belangeri* and *T.b. chinensis* that were closely related to Lagomorpha, whereas Artiodactyla and Perissodactyla diverged after the Scandentia/Lagomorpha cluster. They formed a large evolutionary clade with Primates.

## Discussion and conclusions

This study reports the mitogenome sequence of *T.b. yaoshanensis* for the first time, thus providing fundamental data for further exploration of mtDNA evolution in tree shrews.

## Supplementary Material

Supplemental Material

## Data Availability

The genome sequence data that support the findings of this study are available in GenBank (https://www.ncbi.nlm.nih.gov/) under accession number OP210313. The associated BioProject, SRA, and Bio-Sample numbers are PRJNA870698, SRR21118736, and SAMN30383116, respectively.
