# Supplementary material for: Complete mitochondrial DNA sequence of Tupaia belangeri yaoshanensis (Wang, 1987) from Dayao Mountains in China
Source: Mitochondrial DNA B Resour. 2023 Mar 13;8(3):402–4. doi: 10.1080/23802359.2023.2186723 (PMC10013471; doi:10.1080/23802359.2023.2186723)
Supplement: Supplemental Material [file TMDN_A_2186723_SM1285.docx]

Table S1 Primers for amplification of whole mitochondrial genome of *T.b yaoshanensis*

| **Primers** | **Primer sequence** | **Location** |
| --- | --- | --- |
| **mt1-F** | GTTAATGTAGCTTAAAATAAAG | 1-1599 |
| **mt1-R** | TGGCTGCTTTTAGGCCAACT |  |
| **mt2-F** | GCCTGGTGATAGCTGGTTGT | 1418-3022 |
| **mt2-R** | GGTTGATTAGCGGGTGAGGT |  |
| **mt3-F** | CCTGCTAATCCTGCCCGTAA | 2750-4315 |
| **mt3-R** | AGGATGCATATGGGGGCTAA |  |
| **mt4-1-F** | ACCATAGTAAGCTCGCACTGA | 3947-5031 |
| **mt4-1-R** | ATGTGAGGTTGAGTCTCGCAG |  |
| **mt4-2-F** | TCCCATCACCCAACAACTCC | 4803-5716 |
| **mt4-2-R** | CCGGCTAGTGGTGGGTATAC |  |
| **mt5-F** | CCGGAATAGTCGGGACAGC | 5319-6824 |
| **mt5-R** | GTGGTAAGGAGGAGGGCATC |  |
| **mt6-F** | AGGCTACACCCTTGACCAAAC | 6517-8121 |
| **mt6-R** | GGGCTCATGTTCGTCCTTTG |  |
| **mt7-F** | GGCCTTCCTATCGTAGTCCT | 7958-9572 |
| **mt7-R** | GGGTCAAACCCGCACTCATA |  |
| **mt8-F** | AAGCAGCGGCATGATACTGA | 9299-10874 |
| **mt8-R** | ACCGTAGCCCCCTAGCTTTA |  |
| **mt9-F** | ACCCTAGTAGGCTCACTCCC | 10604-12213 |
| **mt9-R** | GCGTCCGATCGTGAGTATCAT |  |
| **mt10-F** | ACTCTGACCCAAACATTAACCG | 12047-13607 |
| **mt10-R** | TTGTGGCAGGGTGGACTTTA |  |
| **mt11-F** | TCAGGGCAGACCCTATCATCA | 13351-14852 |
| **mt11-R** | GGCCTGATAGAACGGCAAGT |  |
| **mt12-F** | ACCCTTACCCGATTCTTCGC | 14647-16149 |
| **mt12-R** | GTCCGTCGAGCATGGAAAGA |  |
| **mt13-F** | AATCATCAACCCACCCAATA | 15812-16779 |
| **mt13-R** | GGCGAAAAAATTTACTTATG |  |
